# Gender and sex differences in adherence to a Mediterranean diet and associated factors during the COVID-19 pandemic: a systematic review

**DOI:** 10.3389/fnut.2024.1501646

**Published:** 2025-01-07

**Authors:** Gerrit Brandt, Marie Pahlenkemper, Cristina Ballero Reque, Luisa Sabel, Christopher Zaiser, Nora M. Laskowski, Georgios Paslakis

**Affiliations:** University Clinic for Psychosomatic Medicine and Psychotherapy, Medical Faculty, Campus East-Westphalia, Ruhr-University Bochum, Bochum, Germany

**Keywords:** COVID-19, Mediterranean diet, gender differences, sexual and gender minorities, health care inequities

## Abstract

**Background:**

The COVID-19 pandemic has led to significant lifestyle changes, including alterations in dietary habits and increases in sedentary behavior. The Mediterranean diet (MD) has been associated with various health benefits, which are especially important given the health challenges posed by the pandemic. During the pandemic, an overall shift towards consuming more highly processed foods has been observed.

**Methods:**

This systematic review investigated adherence to MD during the COVID-19 pandemic, focusing on gender differences and factors influencing adherence to MD in the general public. The literature search focused on papers published between January 1, 2019, and July 8, 2024, across various databases such as Web of Science (WOS), Scopus, PubMed MEDLINE, and PsycINFO.

**Results:**

Following the PRISMA guidelines, this search identified 14,347 references, of which 5,734 were duplicates. After a thorough multi-level screening process, 29 studies, encompassing 55,242 participants, met the inclusion criteria. While seven studies reported that men adhered to a MD more than women during the COVID-19 pandemic, 12 studies also indicated that women had higher adherence to a MD compared to men. Nine studies, however, found no significant gender differences in MD adherence. Additionally, older age, higher education levels, higher socioeconomic status, and increased physical activity were linked to greater adherence to a MD in the pandemic context.

**Conclusion:**

Gender-specific differences in dietary behavior are influenced by factors such as socioeconomic status, gender roles, and pandemic phases as well as biases in sample composition and methodological weaknesses. Significant gaps in the evidence, particularly concerning sexual and gender minorities, are highlighted.

**Systematic review registration:**

https://www.crd.york.ac.uk/prospero/display_record.php?RecordID=421727.

## Graphical abstract

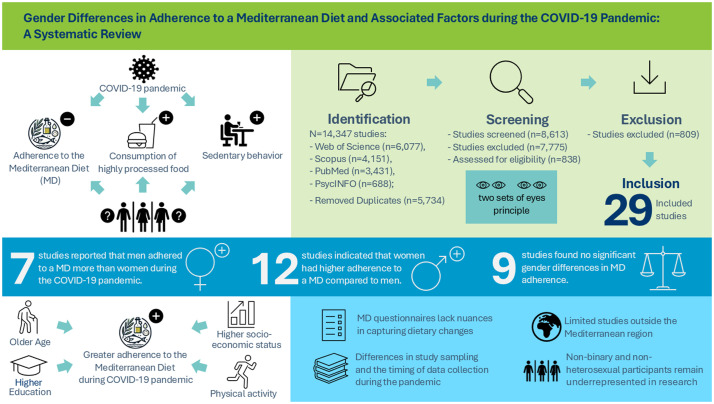

## 1 Introduction

In December 2019, the spread of SARS-CoV-2 was first reported in Wuhan, China, causing the global COVID-19 health crisis with rapid human-to-human transmission and high mortality rates. The World Health Organization (WHO) declared the COVID-19 outbreak a global pandemic in March 2020 (1, 2). Governments and health authorities implemented stringent measures, including lockdowns, social distancing mandates, and home confinements, to control the spread of the disease and reduce the burden on healthcare systems (3). These safety measures have led to significant changes in daily routines and lifestyles, including increases in sedentary behavior and the consumption of highly processed food and sweetened beverages (4, 5). The prolonged times of staying at home, accompanied by increased pandemic-related anxiety, stress, and/or boredom may have contributed to higher levels of emotional eating, leading, in turn, to an increased intake of energy-dense foods and snacks (6). Additionally, the threat of food insecurity may have led to the purchase of more packaged, processed, and longer-lasting foods rather than fresh products, contributing to a further deterioration in diet quality (7).

At the same time, maintaining a healthy diet is crucial to support immune function and overall somatic and mental health. Obesity and diabetes have been identified as important risk factors for the development of a more severe course of COVID-19 (8, 9). In contrast, the Mediterranean diet (MD) has been associated with a reduction in health sequelae of COVID-19, due to anti-inflammatory (10) and anti-thrombotic properties (11, 12). Given these beneficial effects, the WHO has issued nutritional guidelines for the lockdown period, highlighting the key nutritional components of the MD (13, 14).

The MD (15) is a well-researched dietary pattern that encompasses minimally processed foods and low sugars while emphasizing the consumption of whole grains, fruits, vegetables, nuts, seeds, and legumes (16–18). It includes olives and olive oil as primary sources of fat, with dairy products (such as milk, yogurt, and cheese) being consumed moderately. Also, fish is included to a moderate extent, while processed foods, meat, and meat products are only included to a relatively limited degree. The MD has been associated with several health benefits due to its high abundance of foods rich in fiber, glutathione, and further antioxidants while maintaining a balanced ratio of omega-6 to omega-3 essential fatty acids (19–21). The revised “PREvención con DIeta MEDiterránea” study (PREDIMED; *n* = 7,447) (22) found a 31% reduction in cardiovascular risk associated with MD compared to a low-fat diet. Moreover, increased MD adherence has been associated with decreased cardiovascular mortality and the prevention of metabolic syndrome, cancer, and chronic conditions, including obesity (23, 24). However, a recent umbrella review revealed a lack of consistency regarding the relationship between adherence to the MD and various health outcomes, specifically overweight and obesity, musculoskeletal health, inflammation, and cardiometabolic health, emphasizing the necessity for further studies to gain a clearer understanding of these relationships (25). Despite the potential benefits of the MD, Mediterranean societies have been progressively shifting towards a more Westernized diet (26), characterized by high consumption of red meat, processed meat, fried foods (e.g., french fries), refined grains, high-fat dairy products, desserts, and high-sugar drinks (27–29). This shift is likely the result of increased urbanization, globalization and trade liberalization, and lifestyle changes that make processed and calorie-dense foods more accessible and convenient compared to the preparation of meals in the traditional MD (30).

Sex may be an important factor to consider in studies on nutrition, as sex-specific interactions between health parameters and dietary aspects may be influenced by sex differences in hormones, physiology, nutrient metabolism, and the gut microbiome (31). Sex is a complex biological construct encompassing anatomy, physiology, genetics, and hormonal influences (32). Gonadal hormones, such as estrogen and testosterone, exert profound effects on several levels, creating distinct nutritional requirements for men and women (33). Accordingly, sex-related differences in nutritional behavior have been observed, with women typically consuming more plant-based choices and fewer calories due to smaller body size and lower muscle mass compared to men (33). Hormonal fluctuations further affect dietary patterns, such that elevated estrogen levels during the peri-ovulatory period have been associated with reduced food intake; on the other hand, reduced estrogen levels in postmenopausal women have been linked to diminished metabolic flexibility and increased central fat accumulation (34). Additionally, sex modulates metabolic responses, including insulin sensitivity to glucose intake (35). Host metabolism and immunological responses are significantly influenced by the gut microbiome (36), while, in turn, it has been shown that sex, along with body weight and dietary behavior, contributes to shaping the human gut microbiome (37). However, the specific mechanistic interactions between sex and the MD are unclear, and studies examining this relationship are limited (38). Recently, Barrea et al. (39) found that women showed significantly higher adherence to the MD and had lower levels of high-sensitivity C-reactive protein. In the “CORonary Diet Intervention with Olive oil and cardiovascular PREVention” study (40) (CORDIOPREV; *n* = 1,002) a 28% reduction in recurrent major cardiovascular events following a MD versus a low-fat diet was found; the superiority of the MD was more significant among men. It remains unclear whether this effect is due to insufficient statistical power in the women group or if sex is an influential factor in dietary response (40).

Besides sex biology, gender (i.e., socialization and behaviors) and other factors, e.g., age, body weight, educational and socioeconomic status influence food choices and consumption quantities (41, 42). Gender is a multifaceted construct that includes gender identity and expression, along with societal and cultural expectations regarding status, characteristics, and behaviors linked to specific sex traits (32). When comparing diet patterns between men and women, it has been shown that men consume more animal protein and less fruits, while women tend to consume higher amounts of fruits and vegetables but also carbohydrates (41, 43). Such differences in dietary profiles often underly gender-associated differences in concerns around weight and body shape as well as greater concerns about the naturalness of food and ethical issues regarding food found in women (41, 44). However, findings regarding MD-style eating behaviors by gender have remained largely inconclusive, with some studies showing a higher adherence to a MD in women, mostly due to an overall lower consumption of red meat (39, 45). To our knowledge, no studies have yet investigated the adherence to a MD among gender groups beyond men and women, or, more broadly, in sexual and gender minorities (SGM).

Since the COVID-19 pandemic has led to alterations in daily habits, it is important to study how these changes might have affected health-determining variables such as diet quality. Identifying potential gender differences may yield crucial information to policymakers to provide more targeted, gender-specific recommendations and support, including underrepresented groups, e.g., SGM, during periods of crises. Previous systematic reviews have addressed the issue of adherence to a MD—with some notable limitations. For instance, Obeid et al. (46) systematically excluded studies assessing MD adherence during the pandemic, while Moore et al. (47) focused exclusively on individuals with overweight and obesity rather than the general population during the pandemic. Other reviews did not address gender differences (48, 49). Thus, the present systematic review aimed to investigate gender differences in the adherence to a MD in the general adult population during the COVID-19 pandemic. In addition, we aimed to study how variations may have been influenced by different factors, including lifestyle changes brought on by the pandemic.

## 2 Methods

### 2.1 Search strategy

This review was registered in the PROSPERO database (CRD42023421727) (50). The search strategy followed the “Peer Review of Electronic Search Strategies” (PRESS) guideline for systematic reviews and the “Preferred Reporting Items for Systematic Reviews and Meta-Analyses” (PRISMA) guidance (51). The aim was to ensure a comprehensive and evidence-based electronic search. Our search targeted papers published from January 1, 2019, to July 8, 2024, across multiple databases, including Web of Science (WOS), Scopus, PubMed MEDLINE, and PsycINFO. The search query for PubMed is displayed in Table 1 (search terms were modified for adaptation to each database. For the full search strategy of all databases, see Supplementary Table S1).

**Table 1:** Full search string for PubMed.

Topic	Medical subject heading (MeSH)
Eating behavior, body image	((diet[Title/Abstract]) OR (nutrition[Title/Abstract]) OR (binge*[Title/Abstract]) OR (eating behavio*[Title/Abstract]) OR (eating habit*[Title/Abstract]) OR (eating disorder*[Title/Abstract]) OR (disordered eating[Title/Abstract]) OR (body *satisfaction[Title/Abstract]) OR (body image[Title/Abstract])) AND
Sex and gender	((gender*[Title/Abstract]) OR (sex[Title/Abstract]) OR (men[Title/Abstract]) OR (women[Title/Abstract]) OR (trans*[Title/Abstract]) OR (lgb[Title/Abstract]) OR (lgbt*[Title/Abstract]) OR (intersex*[Title/Abstract]) OR (*binary[Title/Abstract]) OR (queer[Title/Abstract]) OR (male[Title/Abstract]) OR (female[Title/Abstract])) AND
COVID-19	((sarscov[Title/Abstract]) OR (pandem*[Title/Abstract]) OR (COVID-19[Title/Abstract]) OR (corona*[Title/Abstract]) OR (lockdown[Title/Abstract]) AND
Language	((English[Language]) OR (German[Language])) AND
Time	(2019:2024[pdat]))

The asterisk (*) serves as a wildcard to substitute for 0 or more characters, capturing various word variations (e.g., “behavio*” includes “behavior,” “behaviour,” “behavioral” etc.).

### 2.2 Selection of primary studies

The selection, management, and organization of the primary studies were conducted using the Covidence software (52). The study selection followed a two-stage “two sets of eyes principle” procedure to ensure thoroughness and minimize subjective bias. In the first stage, each article’s title and abstract were independently reviewed by two authors from the review team (MP, CR, LS, CZ, and NL). In the second stage, the full texts of the selected articles were again screened by two out of six independent authors (GB, MP, CR, LS, CZ, and NL). The pairs of authors alternated randomly throughout the process to reduce potential bias. Any conflicts or inconsistencies were resolved through discussion within the team. The final selection of studies was approved by all authors. The synthesis of outcomes was carried out by GB.

### 2.3 Inclusion and exclusion criteria

We included original research articles written in English or German language with no geographical limitations. We included studies carried out in the general adult population without clinical diagnoses, regardless of gender, and excluded populations of children, adolescents (<18 years), and clinical populations. We also excluded systematic reviews, reviews, meta-analyses, case reports, qualitative studies, commentaries, conference papers, opinion pieces, letters, editorials, articles written in languages other than German or English, and publications before 2019 and/or without an explicit COVID-19 reference. Only studies reporting outcomes regarding both a MD as well as gender-specific analyses and/or SGM populations (including but not limited to lesbian, gay, bisexual, pansexual, transgender, non-binary, queer, two-spirit, intersex, and asexual individuals) were included, whereas studies regarding other outcomes (e.g., disordered eating behaviors, body image concerns) and studies without gender comparisons were excluded. All outcome effect measures were included.

### 2.4 Quality assessment

As suggested by Ma et al. (53), the quality assessment was performed using the “Critical Appraisal Checklist for Analytical Cross-Sectional Studies” (54). The checklist comprises eight items assessed as “Yes,” “No,” “Unclear,” and “Not Applicable,” and an overall appraisal rated as “Include,” “Exclude,” or “Seek Further Info.” The quality assessment of the included studies was independently carried out by two authors out of the author team (GB, MP, CR, LS, CZ, and NL), with the first author (GB) making the final assessment decision.

## 3 Results

### 3.1 Study extraction

**Figure 1:**
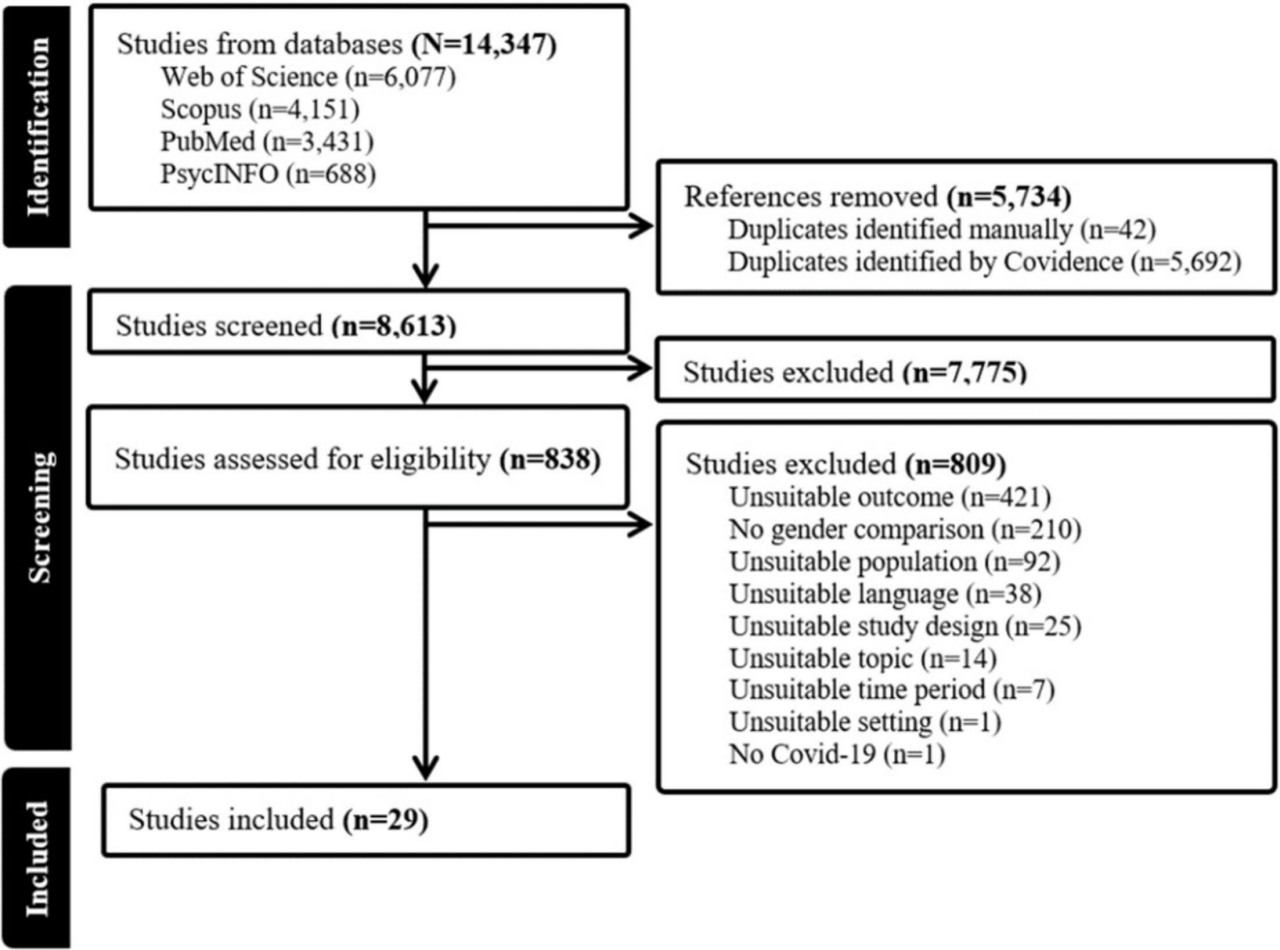
PRISMA diagram.

The literature search yielded 14,347 results. In the end, 29 studies were selected for this synthesis (see PRISMA diagram in Figure 1).

**Figure 2:**
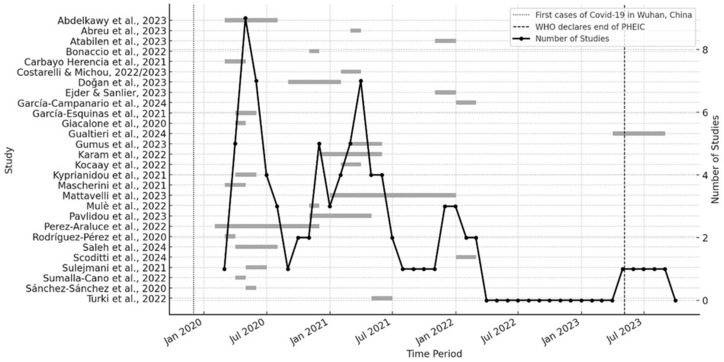
Survey periods and number of included studies over time.

Twenty-five out of the 29 studies were cross-sectional. Among these, 17 retrospectively assessed self-reported changes in MD habits during the pandemic. Four studies conducted measurements at two time points (see Figure 2). In 15 of the included studies, the data collection was conducted in 2020; nine studies were conducted in 2021 and five in 2022 or later (see Figure 2). Two publications reported results based on the same sample (55, 56).

### 3.2 Study characteristics

The cumulative sample size in all 29 included studies amounted to a total of 55,242 participants. Sample sizes ranged from *n* = 168 to *n* = 9,413 (men: 56–3,549; women: 111–5,864). The gender distribution in the studies was notably skewed, with 18,999 men (34.39%) and 36,209 (65.55%) women. Only three studies reported including participants who identified as neither men nor women, but without providing further details (57–59); these participants accounted for less than 0.06% of the cumulative sample. The terms “sex” and “gender” in this review are used in alignment with their usage in the original studies included in this synthesis. There were no results reported for this group. Twelve studies had predominantly younger participants (>50% of the sample <39 years), while five studies assessed predominantly older participants (>50% of the sample >50 years; see Table 2). Twenty-two studies, thus the majority of the included studies recruited samples with high levels of education, predominantly consisting of university-educated participants. Seven studies included samples in Spain, six in Italy, five in Turkey, two in Egypt, two in Greece, and one each in Cyprus, Denmark, Kosovo, Lebanon, Portugal, and Tunisia. Apart from Denmark, all countries are considered to be Mediterranean.

**Table 2:** Gender differences in Mediterranean diet patterns.

Author, year	Measure	Country	Sample			Result regarding gender differences
			*N* (women %)	Age (years)	Education	
Abdelkawy et al., 2023 (87)	MEDAS^a^ (modified^c^), 1 time point	Egypt	*N* = 1,010 (77%)	≤20: 8.6% 21–35: 67.6% 36–50: 21.7% >50:2.1%	University: 62.3% Postgraduate: 31.2% Secondary: 6.5%	Significant changes in daily intake of vegetables, butter, margarine/cream, honey, fish/seafood, cooked vegetables, pasta, rice between men and women. However, the authors did not report the direction of the effects Women significantly increased their intake of homemade pastries compared to men (39%, *p* < 0.05) Men significantly increased intake of carbonated beverages (21%, *p* < 0.05) compared to women
Abreu et al., 2023 (86)	MEDAS, 1 time point	Portugal	*N* = 1,114 (67.7%)	Mean: 19.10 SD: 1.72	University students: 100%	No significant gender differences in adherence to MD Women exhibited higher MEDAS scores regarding olive oil use (*p* = 0.049); consumption of fruit (*p* = 0.011); red meat, hamburger, or meat products (*p* < 0.001); butter, margarine, or cream (*p* < 0.001); carbonated beverages (*p* = 0.012); fish/seafood (*p* = 0.010), and preferring white meat over red meat (*p* < 0.001)
Atabilen et al., 2023 (79)	MEDAS, 1 time point	Turkey	*N* = 6,609 (69.8%)	Range: 18–70 Women: Median: 22.0 IQR: 8.0 Men: Median: 23.0 IQR: 9.06	Basic literacy: 1% Primary school: 3% Secondary school: 3% High school: 16% University graduates: 73% Postgraduate: 4%	Significant gender differences in adherence to MD (*p* < 0.001) Men more often exhibited low adherence (38.8%) than women (26.3%) Women more often showed moderate (64.6%) and high adherence (9.1%) than men (52.8 and 8.4%)
Bonaccio et al., 2022 (65)	MedCOVID-19^a^, 1 time point	Italy	*N* = 4,400 (57.7%)	65–75: 54.7% >75: 45.3%	Up to lower secondary: 53.0% Upper secondary: 37.0% Postgraduate: 10.0%	Women had a higher risk of having decreased adherence to MD compared to men [OR = 1.86; 95% CI = (1.58–2.21)] Men and women increased their consumption of fruit and nuts, vegetables, and legumes, and decreased consumption of meat and meat products during the COVID-19 pandemic Men increased alcohol consumption more than women (*p* = 0.027)
Carbayo Herencia et al., 2021 (66)	MEDAS (modified^d^), 2 time points	Spain	*N* = 207 (66.2%)	Mean: 51.3 SD: 12.4 Range: 20–83	Primary, secondary, or vocational training: 15.0% Diploma or degree: 65.2% Doctorate: 19.8%	Men scored higher regarding adherence to MD compared to women (*p* < 0.05) Men consumed significantly more wine and pulses per week than women (*p* ≤ 0.001) Increase in adherence to MD observed across the sample during lockdown with no significant differences by gender (increase mainly explainable by increased consumption of olive oil, fish, and seafood)
Costarelli and Michou, 2022 (55) and Costarelli and Michou, 2023^b^ (56)	MEDAS^a^, 1 time point	Greece	*N* = 2029 (75.3%)	Mean: 38.90 SD: 11.31	Up to high school: 15.10% Tertiary education: 56.30% MSc/PhD: 28.50%	The majority of both men (61.1%) and women (70.4%) showed moderate adherence to the MD Men and women significantly differed in MEDAS categories (*p* < 0.001), with 23.6% of men showing low adherence to the MD compared to 15.7% of women Total adherence to MD was greater in women (*p* = 0.037)
Doğan et al., 2023 (74)	MEDAS^a^, 1 time point	Turkey	*N* = 949 (66.4%)	Mean: 27.1 SD: 8.2 Range: 18–59	Non-educated: 2.0% Primary school: 5.7% High school: 32.0% University: 60.3%	Medium and higher adherence to MEDAS was reported more frequently in women (medium = 33.3%; high = 20.8%) compared to men (medium = 26.3%; high = 13.2%) Men were more likely to show low adherence to MEDAS (60.5%) compared to women (45.9%)
Ejder and Sanlier, 2023 (80)	MEDAS, 1 time point	Turkey	*N* = 1,117 (77.9%)	Mean: 27.2 SD: 10.44 Range: 19–65 19–39: 72.7% 31–65: 27.3%	*<*High school graduate: 10.6% University graduate: 89.4%	No significant difference between genders regarding MEDAS score (*p* = 0.067)
García-Campanario et al., 2024 (59)	MEDAS, 1 time point	Spain	*N* = 303	Mean: 21.48 SD: 4.57 Women: Mean: 20.93 SD: 2.73 Men: Mean: 21.8 SD: 5.34	University students: 100%	No significant differences between genders regarding MEDAS score Women consumed less olive oil (*p* = 0.0071) and greater amounts of red meat or processed meat (*p* = 0.0031), and chicken, turkey, or rabbit (*p* = 0.033) compared to men
García-Esquinas et al., 2021 (81)	MEDAS, 2 time points	Spain	*N* = 3,041 (57.7%; varying across cohorts: 49.5–78.8%)	Mean: 74.5 (varying means across cohorts and time points: 69.9–81.5)	University studies: 8.0–22.3%	No significant association between gender and changes in MEDAS scores was observed between the periods before and during the confinement
Giacalone et al., 2020 (58)	MEDAS^a^, 1 time point	Denmark	*N* = 2,462 (71.1%)	18–35: 35.3% 36–50: 37.2% 51–65: 23.5% >65: 3.9%	Bachelor and above: 88.5% High school and vocational training: 10.8% Basic education: 0.3% Other: 0.4%	Women had slightly higher adherence to MD than men (mean MEDAS score: 5.6 vs. 5.1, *p* < 0.001) Men and women differed in their consumption of vegetables, fruit, carbonated beverages, legumes, fish, commercial pastries, homemade pastries, and alcohol, but the effect sizes were small Women exhibited a higher increase in the consumption of commercial pastry (*p* = 0.001), homemade pastry (*p* < 0.001), and alcohol (*p* = 0.037) compared to men
Gualtieri et al., 2024 (73)	MEDAS^a^, 1 time point	Italy	*N* = 776 (71.26%)	Mean: 43.1 SD: 17.96 Women: Mean: 42.8 SD: 18.36 Men: Mean: 43.84 SD: 16.93	n.r.	No significant differences between genders regarding MEDAS score: 9.4% of women and 14.35% of men exhibited low adherence; 62.57% of women and 61.43% of men exhibited medium adherence; 28.03% of women and 24.22% of men exhibited high adherence to MD Women showed higher adherence regarding the consumption of olive oil (*p* < 0.01), vegetables (*p* < 0.001), red meat (*p* < 0.011), and white meat (*p* < 0.001) compared to men Men exhibited higher adherence regarding the consumption of wine (*p* < 0.001) and legumes (*p* < 0.014) compared to women
Gumus et al., 2023 (75)	MEDAS^a^, 1 time point	Turkey	*N* = 820 (76.6%)	Mean: 31.3 SD: 10.3 Range: 18–65	n.r.	Women reported significantly higher levels of adherence to MD (MEDAS) compared to men (*p* < 0.001) Women reported greater increases during the pandemic in the consumption of dry fruits, fresh vegetables, pasta and grains, whole grain foods, milk and yogurt, cheese, fish, legumes, white meat, homemade food, and homemade pastries, and lower intake in fast food and takeaway food compared to men (*p* < 0.05)
Karam et al., 2022 (67)	MEDAS, 1 time point	Lebanon	*N* = 1,030 (67,5%)	18–25: 51.5% 26–44: 38.1% 45–70: 10.5%	High school or less: 16.5% University student: 51.2% University graduate: 32.3%	Men exhibited marginally higher mean adherence to MD than women Men reported a higher intake of fruits, wine, fish and fish products, and nuts, whereas women consumed more legumes and sofrito, and preferred chicken over other meat (*p* < 0.05) No significant differences were found between genders regarding the consumption of olive oil, fruits, red meat, butter/margarine, sugary drinks, and processed desserts
Kocaay et al., 2022 (76)	MEDAS^a^, 1 time point	Turkey	N = 562 (70.3%)	Mean: 23.81 SD: 1.11 Range: 22–26	Medical students: 100%	MEDAS score was significantly higher in women than men (p < 0.001)
Kyprianidou et al., 2021 (68)	MedDietScore^a^, 1 time point	Cyprus	N = 1,485 (60%)	Mean: 35.8 SD: 12 18–24: 18.9% 25–44: 58.1 > 45: 23.1	Primary education: 0,3% Middle or high school: 17.4% University degree: 82.3%	More women (36%) than men (29%) showed low adherence to MD; more men (48%) than women (52%) showed high adherence to MD (p = 0.02) Gender was not a significant predictor for the level of MD adherence
Mascherini et al., 2021 (69)	MedDietScore^a^, 2 time points	Italy	*N* = 1,383 (72.6%)	Women: Mean: 29.8 SD: 12.2 Men: Mean: 33.2 SD: 14.5	Secondary school: 0.3% High school: 53.9% University: 36.3% PhD: 9.5%	Being a man was associated with adherence to MD [*b* = 0.16, 95% CI = (0.14–0.46)]
Mattavelli et al., 2023 (70)	MEDAS, 2 time points	Italy	*N* = 711 (58%)	Mean: 68.1 SD: 10.0	n.r.	No significant gender differences in MEDAS score at baseline After a 4.5-year follow-up, significantly higher reductions in MEDAS scores in women (−5%) compared to men (−2%, *p* < 0.001) were found No significant gender differences between individuals who improved and who deteriorated in MEDAS scores from baseline to follow-up (*p* = 0.12) From baseline to follow-up, the consumption of sofrito but also commercial sweets or pastries, and butter, margarine, or cream increased in men and women From baseline to follow-up, greater reduction in olive oil use in women (−60%) compared to men (−37%), and a larger decrease in weekly nut consumption in women (−45% compared to −24% in men) was found
Mulè et al., 2022 (82)	KIDMED, 1 time point	Italy	*N* = 533 (62.8%)	Mean: 21.46 SD: 0.18	University students: 100%	No significant gender differences in adherence to MD Significant relationship between gender and the consumption of beer (OR = 0.365, *p* < 0.001) and wine (OR = 0.659, *p* = 0.027), with a lower consumption in women
Pavlidou et al., 2023 (78)	MedDietScore^a^, 1 time point	Greece	*N* = 3,721 (50.5%)	Mean: 37.6 SD: 5.8 Range: 21–65	Mean: 12.2 years of education SD: 2.8	Women more frequently exhibited moderate (61.9%) and high (62.6%) adherence to MD compared to men (38.1, 37.4%), and were less likely in the low (32.5) and very low (46.2%) MD compliance quartile compared to men (68.5, 53.8%; *p* < 0.001) Women exhibited a 35% higher probability of greater compliance to MD compared with men (*p* = 0.009)
Perez-Araluce et al., 2022 (83)	MDS, 1 time point	Spain	*N* = 9,413 (62.3%)	Mean: 52.6 SD: 12.4	Years of university education: Median: 5 IQR: 4–5	No significant gender differences in adherence to MD (*p* = 0.471)
Rodríguez-Pérez et al., 2020 (77)	MEDAS^a^, 1 time point	Spain	*N* = 7,509 (70.6%)	<20: 3.0% 21–35: 34.0% 36–50: 31.6% 51–65: 25.7% >65: 5.7%	University: 46.4 Postgraduate: 31.5 Professional: 13.9 Primary: 8.2	Significant gender differences in the level of adherence to the MedDiet (low, medium, high) with more women reporting a higher adherence compared to men (*p* < 0.001)
Saleh et al., 2024 (85)	MEDAS^a^ (modified^c^), 1 time point	Egypt	*N* = 306 (70%)	20–35: 83% 35–50: 2.9% 50: 1.3%	Postgraduate: 17% Secondary or less: 7.8% Bachelor: 75%	Gender did not significantly affect changes in food consumption Decrease in soft drink consumption in men (46.7%) and women (46.3%; n.s.) Increase in consumption of fruits and vegetables in men (31.5%) and women (45.8%; n.s.)
Sánchez-Sánchez et al., 2020 (4)	MEDAS^a^, 1 time point	Spain	*N* = 1,065 (72.8%)	Mean: 38.7 SD: 12.4 16–25: 17.9% 26–40: 39% 41–55: 32.9% 56–70: 9.8% ≥71: 0.4%	n.r.	No statistically significant differences between genders before or during pandemic-related confinement measures
Scoditti et al., 2024 (72)	MEDAS^a^, 1 time point	Italy	*N* = 748 (57.6%)	≤39: 12.0% 40–49: 36.8% 50–59: 38.1% 60: 13.1%	With graduation: 82.1% No graduation: 17.9%	Women reported higher adherence to MD than men (*p* < 0.003) while working from home Variation concerning pre-pandemic assessments did not depend on gender (*p* = 0.06)
Sulejmani et al., 2021 (57)	MEDAS^a^, 1 time point	Kosovo	*N* = 689 (71%)	18–35: 71% 36–50: 19% ≥51: 10%	University: 88% Lower: 12%	Women exhibited higher odds of a change in their adherence to MD during the lockdown (OR: 5.17), representing a higher adherence compared to men
Sumalla-Cano et al., 2022 (71)	MEDAS^a^, 1 time point	Spain	N = 168 (66.7%)	<20 years: 32.1% 21–35 years: 47.6% >36 years: 20.2%	University students: 76.8% Research Professors: 14.9% Administrative Staff (8.3%)	Medium/High adherence to MD during confinement was highly frequent in men (82.1%) and women (77.6%) Low adherence to MD in 22.3% of women and 17.8% of men Significantly higher consumption of fish and seafood (15.9%) in women compared to men (*p* < 0.05) Higher increase in the number of consumed meals per day in women compared to men (17.9%, *p* = 0.077)
Turki et al., 2022 (84)	MEDAS, 1 time point	Tunisia	*N* = 1,082 (74.3%)	Mean: 32.5 SD: 12.0 20–25: 31.3% 25–60: 63.9% ≥60: 4.8%	Not graduated: 0.4% Primary school: 0.8% Secondary school: 7.0% University: 91.8%	No significant gender differences in mean MEDAS score (*p* = 0.24) Equal distribution of adherence to MD categories between men and women (*p* = 0.433)

MEDAS, Mediterranean Diet Adherence Screener; MedCOVID-19, Mediterranean COVID-19 Pandemic Score; MedDietScore, Mediterranean Diet Score; KIDMED, Mediterranean Diet Quality Index in children and adolescents; MDS, Mediterranean Diet Score; OR, odds ratio.

a Retrospective self-report of pre-pandemic and pandemic nutritional behavior.

b The studies by Costarelli and Michou (55) and Costarelli and Michou (56) refer to the same sample and are therefore combined in this table.

c Wine consumption was excluded due to the low general intake in Egypt, as alcohol is prohibited in Islamic countries.

d The question on wine intake was excluded due to ethical concerns, as the question was considered to potentially encourage greater alcohol consumption during the lockdown phase. Please refer to the main text for more details on questionnaires and study characteristics.

### 3.3 Questionnaires

Twenty out of the 29 studies used the Mediterranean Diet Adherence Screener (60, 61) (MEDAS) for assessing adherence to a MD, and two studies used modified versions of the MEDAS, excluding the alcohol-related items. Three studies used the Mediterranean Diet Score (MedDietScore) by Panagiotakos et al. (62), and one study used the Mediterranean Diet Score (MDS) by Trichopoulou et al. (63). One study used the Mediterranean Diet Quality Index in children and adolescents (KIDMED) (64), and another study assessed adherence to a MD using the Mediterranean COVID-19 Pandemic Score (MedCOVID-19)—a self-designed questionnaire (65). Please see Table 2 for more details.

The MEDAS questionnaire (60, 61) includes 12 questions on the consumption frequency of certain food categories and two questions on food intake habits characteristic of the MD, each scored zero or one. Points are given for high consumption of favorable and limited consumption of unfavorable foods. One point is given for the following: using olive oil as the main cooking fat, preferring white meat (i.e., chicken) over red meat (i.e., pork and beef), consuming more than four tablespoons of olive oil daily, consuming more than two servings of vegetables daily, eating more than three portions of fruit daily, eating more than three servings of pulses and fish weekly, consuming at least three servings of nuts weekly, or two or more servings of sofrito weekly, eating a maximum of one serving of red meat, or sausages, or animal fat daily, consuming less than 100 mL of sugar-sweetened beverages daily, drinking less than seven glasses of red wine weekly, and eating less than three portions of commercial sweets or pastries weekly. If a condition is not met, it receives a score of zero, resulting in a total score ranging from zero to 14.

The MedDietScore (62) is a questionnaire for assessing eating habits based on the frequency of weekly consumption of specific food groups more or less aligning with the MD. The nine primary food groups evaluated are non-refined cereals (e.g., whole grain bread, pasta, brown rice), fruits, vegetables, legumes, potatoes, fish, meat and meat products, poultry, and full-fat dairy products (e.g., cheese, yogurt, milk). Additionally, olive oil and alcohol are included as separate categories. Each food group receives a score according to its alignment with the MD. The total score ranges from zero to 55, with higher values indicating greater adherence to the MD.

The MDS (63) is based on the consumption levels of nine foods: vegetables, legumes, fruit, nuts, whole grains, red/processed meat, fish, alcohol, and the monounsaturated to saturated fat ratio. A score of zero is given, if the intake of positive components (vegetables, legumes, fruit, nuts, whole grains, fish, and the fat ratio) is below the median of the norm population, and a score of one if it is equal to the median or above it. For red/processed meat, a score of one is assigned for consumption below the population median, and zero for above it. The total score for the MDS can range from zero, indicating minimal adherence, to nine, indicating perfect MD adherence.

The KIDMED questionnaire (64) analyzes adherence to the MD through 16 questions, resulting in a final score of zero to 12. The total score indicates three levels of adherence: 0–3 for poor adherence, 4–7 for medium adherence, and 8–12 for high adherence to the MD.

In the self-report measure MedCOVID-19 (65), points are assigned based on dietary and lifestyle changes during the pandemic. Positive points (+1) are given for increased consumption of recommended foods from the MD (i.e., fruits, vegetables, legumes, cereals, fish, and olive oil) and corresponding favorable behaviors (i.e., local/organic food, physical exercise), while negative points (−1) are assigned for decreased consumption and a decrease in favorable behaviors during the pandemic. The total score ranges from −14 to 14, with higher scores indicating higher adherence to MD-style eating habits during the pandemic.

### 3.4 Gender differences

#### 3.4.1 Overall adherence

The results of the included studies regarding gender differences in adherence to a MD are heterogeneous. Seven studies reported that compared to women, men showed higher scores of adherence to a MD during the pandemic (65–71). Specifically, in the longitudinal study conducted by Mattavelli et al. (70), which included a pre-pandemic baseline, women exhibited a significantly greater decline in adherence to a MD, showing a reduction of 5% compared to a 2% reduction in men over a 4.5-year follow-up period (*p* < 0.001) (70). In contrast, 12 studies indicated that women demonstrated greater adherence to a MD during the pandemic compared to men (55–58, 72–79). Finally, nine studies reported no significant gender differences regarding adherence to a MD during COVID-19 (4, 59, 80–86).

Seven out of 12 studies reporting that women adhered to a MD to a greater degree than men had samples with more than 70% women (56–58, 73, 75–77). Studies reporting higher adherence to a MD in men had more gender-balanced samples, except for one study (69). All but one study reporting higher adherence among women used the MEDAS questionnaire and was cross-sectional, with all studies but two applying retrospective data collection to determine the impact of the pandemic.

Three out of the four studies that collected data at two time points reported higher adherence to a MD among men (66, 69, 70). Three of the studies with longitudinal design had (relatively) older samples (66, 70, 81), two had Spanish samples (66, 81), and two had Italian samples (69, 70). One of those studies had a sample with more than 70% women (69). One study provided data solely at the item level and did not include further information on adherence to a MD (87). The results regarding gender differences in adherence to a MD are summarized in Table 2.

#### 3.4.2 Adherence to food categories

Eleven studies reported gender differences at an item level (i.e., food category level) in the questionnaires assessing the adherence to a MD during the pandemic (58, 59, 65–67, 70, 71, 73, 75, 86, 87). These studies highlight women’s dietary preferences for certain foods, despite the findings not being unequivocal. For example, women tended to consume more olive oil than men in two studies (73, 86), but the reduction in olive oil use in women compared to men during the pandemic was greater in another study (70). Women favored a higher intake of vegetables (73, 75) and showed a greater preference for fruits (75, 86). The consumption of sofrito was also higher in women compared to men (67). Moreover, women consumed more legumes (67, 75) and more frequently preferred white meat over other meat, with five studies confirming this observation (59, 67, 73, 75, 86). On the other hand, nut consumption was less prevalent in women than in men (70). Lastly, during the pandemic, women were reported to consume more fish and seafood (71) as well as whole grain foods, milk and yogurt, cheese, fish, and homemade food (75). Contrary to these findings, a study found men to consume more fruits, nuts, and fish (products) than women (67); furthermore, one study found men to consume more pulses than women (66).

Regarding unfavorable food categories, two studies showed that commercial sweets or pastries consumption (70, 85), but also the consumption of butter, margarine, or cream (70) increased both in men and women during the pandemic. Three studies showed that women consumed significantly more (homemade) pastries than men (58, 75, 87). Women displayed a more moderate consumption of red meat, hamburgers, or other meat products (73, 86) as well as butter, margarine, cream, and carbonated beverages (86). Men, by contrast, were found to consume more carbonated beverages (87).

Three studies reported that men increased their alcohol consumption compared to women (65–67). Additionally, one study reported a lower consumption of beer and wine in women (82). However, in a Danish sample, women increased their consumption of alcohol during the pandemic to a higher extent than men (58). Gualtieri et al. (73) found men to exhibit a higher adherence regarding a moderate consumption of wine compared to women.

### 3.5 Factors influencing changes in adherence to the Mediterranean diet during COVID-19

Nineteen studies explored factors influencing changes in adherence to a MD during the COVID-19 pandemic. Of these, eight studies identified factors linked to a decrease in adherence to a MD, such as overweight/obesity (76, 78) and low physical activity (77). Furthermore, 13 studies highlighted factors associated with an increase in adherence, including higher education (57, 65–67, 77) and socioeconomic status (65). The most robust evidence for a decline in adherence to a MD was present for overweight/obesity and smoking. The most robust evidence for an increase in adherence to a MD included physical activity, higher education, higher economic status, not living alone, and living in a family home. The relationship between age and changes in dietary adherence was examined in 11 studies, yielding conflicting evidence. Table 3 provides a summary of published factors influencing changes in adherence to a MD during COVID-19. These factors were not examined by gender in any of the studies.

**Table 3:** Factors influencing changes in adherence to the Mediterranean diet during COVID-19.

Factors associated with a *decrease* in adherence to a MD during COVID-19	Factors associated with an *increase* in adherence to a MD during COVID-19	Factors with *contradictory* evidence regarding adherence to a MD during COVID-19
Having a PhD (57) Lower physical activity (77) Overweight/obesity (76, 78) Current smoking (68, 78) Being unmarried, divorced, or widowed (68) Having children in care (77) Living in areas with more than 50,000 inhabitants (65) Having one or more daily meals out of home (77) General anxiety (78) Anxiety associated with COVID-19 pandemic (74) Getting up/sleeping late (82)	Higher education (57, 65–67, 77) Studying ecotrophology (71) Higher socioeconomic status (65) Higher occupational status (65, 84) Higher income (67) Higher household welfare (78, 84) Living in rural areas (78) Physical activity (67, 68, 77, 78, 81) Lower body mass index (58) Living in a family home/not alone (57, 71, 78) Having no daily meal out of home (87) Better general health (81) Moderate consumption of alcohol (81) Lower intake of fried foods, alcohol, fast food, and snacks (77) Waking up early and going to bed early (82) Adequate sleep quality (78)	Older age: Increase (58, 67, 77, 79, 80, 84, 87) Decrease (65, 70, 81) Younger age: Increase (69) Decrease (87)

MD, Mediterranean diet.

### 3.6 Quality assessment

The quality assessment revealed variations across the included studies, with most studies meeting key criteria such as clearly defined sample inclusion, detailed descriptions of study subjects and settings, identification and management of confounding factors, valid and reliable outcome measurements, and appropriate statistical analysis. Certain studies, nonetheless, demonstrated deficits in specific methodological aspects (see Table 4).

**Table 4:** Quality assessment of the included studies.

First author, year	Were the criteria for inclusion in the sample clearly defined?	Were the study subjects and the setting described in detail?	Were confounding factors identified?	Were strategies to deal with confounding factors stated?	Were the outcomes measured in a valid and reliable way?	Was appropriate statistical analysis used?
Abdelkawy et al., 2023 (87)	Yes	Yes	?	No	Yes	?
Abreu et al., 2023 (86)	Yes	Yes	No	No	Yes	Yes
Atabilen et al., 2023 (79)	No	Yes	No	Yes	Yes	Yes
Bonaccio et al., 2022 (65)	?	Yes	Yes	Yes	Yes	Yes
Carbayo Herencia et al.,2021 (66)	No	Yes	Yes	No	Yes	Yes
Costarelli and Michou, 2022 (55)	Yes	Yes	Yes	No	Yes	Yes
Costarelli and Michou, 2023 (56)	Yes	Yes	?	No	Yes	Yes
Doğan et al., 2023 (74)	Yes	Yes	?	No	Yes	Yes
Ejder and Sanlier, 2023 (80)	Yes	Yes	Yes	Yes	Yes	Yes
García-Campanario et al., 2024 (59)	Yes	Yes	No	No	Yes	Yes
García-Esquinas et al., 2021 (81)	Yes	Yes	Yes	?	Yes	Yes
Giacalone et al., 2020 (58)	Yes	Yes	?	No	Yes	Yes
Gualtieri et al., 2024 (73)	Yes	Yes	Yes	Yes	Yes	Yes
Gumus et al., 2023 (75)	Yes	Yes	No	No	Yes	Yes
Karam et al., 2022 (67)	Yes	Yes	Yes	?	Yes	Yes
Kocaay et al., 2022 (76)	No	Yes	Yes	Yes	Yes	Yes
Kyprianidou et al., 2021 (68)	Yes	Yes	Yes	Yes	Yes	Yes
Mascherini et al., 2021 (69)	Yes	Yes	Yes	?	Yes	Yes
Mattavelli et al., 2023 (70)	?	No	Yes	No	Yes	Yes
Mulè et al., 2022 (82)	?	Yes	Yes	?	Yes	Yes
Pavlidou et al., 2023 (78)	Yes	No	Yes	Yes	Yes	Yes
Perez-Araluce et al., 2022 (83)	Yes	Yes	Yes	Yes	Yes	Yes
Rodríguez-Pérez et al., 2020 (77)	Yes	Yes	No	No	Yes	Yes
Saleh et al., 2024 (85)	Yes	Yes	Yes	No	No	Yes
Sánchez-Sánchez et al., 2020 (4)	Yes	Yes	Yes	Yes	Yes	Yes
Scoditti et al., 2024 (72)	Yes	Yes	Yes	Yes	Yes	Yes
Sulejmani et al., 2021 (57)	Yes	Yes	?	?	Yes	Yes
Sumalla-Cano et al., 2022 (71)	No	Yes	Yes	No	Yes	Yes
Turki et al., 2022 (84)	Yes	Yes	Yes	Yes	Yes	Yes

? = Unclear. Since the exposure condition in the present review was the COVID-19 pandemic, the aspect” Was the exposure measured in a valid and reliable way?” was omitted from the quality assessment. Similarly, as the review focused on the general population and excluded clinical groups, the question” Were objective, standard criteria used for measurement of the condition?” was also omitted.

## 4 Discussion

This systematic review aimed to synthesize studies dealing with the MD, identify potential gender differences and examine factors influencing the adherence to a MD during the COVID-19 pandemic. Following a thorough and comprehensive methodical approach, the synthesis revealed heterogeneous results regarding gender differences, with studies showing higher adherence to a MD in either men or women, or no differences.

Several factors could account for these results. To begin with, studies reporting greater adherence in women predominantly had samples with more than 70% of women participants; thus, disparities in sample gender compositions may mask true gender differences. In contrast, studies with a more balanced gender distribution showed higher adherence to a MD in men. Moreover, a potential selection bias due to non-random sampling (snowball- or convenience sampling) could have influenced the observed gender differences in adherence to a MD during the COVID-19 pandemic. For example, Wild et al. (88) demonstrated that undergraduate students, who are popular survey groups, are not representative of the general population or age-matched non-students. Consequently, research in representative and randomly selected cohorts is asked for. The timing of the data collection during the pandemic may also be a relevant aspect to consider; studies indicating higher adherence in men were more frequently conducted during the early phase of the COVID-19 pandemic in 2020, whereas five of the eight studies showing greater adherence in women were conducted in the later phases of the pandemic. While the pandemic has impacted everyone, it has generally posed greater challenges for women, who have experienced an increased risk of infection and psychological stress (89). At the same time, women tend to use a more emotion-focused coping style, including emotional eating (90, 91). Women were found to exhibit more stress and emotional eating during the pandemic as well (92, 93). As the pandemic progressed and situations adapted to the “new normal,” women might have adopted or maintained better dietary habits (94), facilitated by the stabilization of daily routines and a re-orientation towards a health and well-being focus. These assumptions suggest that a pandemic’s progression might significantly impact everyone’s dietary habits, but that men and women may respond to such crises differently over time, which also warrants investigations. Our systematic search also yielded nine studies that reported no significant gender differences in adherence to a MD. However, the absence of statistically significant differences does not necessarily imply a true lack of effects between genders. Instead, it may reflect limitations such as insufficient statistical power, variability in sample sizes, or heterogeneity in study designs (95). These factors may have obscured potential small but meaningful differences between genders and highlight the complexity of interpreting non-significant findings. Future studies should also aim for larger, more homogenous samples and robust study designs to better detect and understand potential gender differences in MD adherence.

Adherence to a MD is generally positively correlated with income (96). Studies have shown that individuals with higher incomes tend to eat healthier foods, regardless of gender (96–98), which facilitates adherence to a MD. Aytekin Sahin and Mengi Celik (99) recently indicated that income positively influenced adherence to a MD in Turkish women (*n* = 2,675). In countries with a more rigid (“traditional”) distribution of gender roles, women often take on the primary role of managing food purchases and meal preparation, perhaps adhering more to healthier diets, while men are less involved in food preparation and may prioritize other aspects of life over diet, resulting in lower adherence to a MD (100). Interestingly, five of the eight studies reporting a higher women’s adherence were conducted in less affluent countries, specifically four in Turkey (per capita GDP, 2021: US$9,654) (101) and one in Kosovo (per capita GDP in 2021: US$5,320) (101). In contrast, only two of seven studies reporting higher adherence in men were from less affluent countries, namely Egypt (per capita GDP in 2021: US$4,150) (101) and Lebanon (per capita GDP in 2021: US$3,040) (101). All of the above implies that, while income generally facilitates healthier eating habits, gender roles could modulate this relationship. Again, further studies are necessary to dissect these complex interactions and better understand how economic factors and gender roles may jointly influence dietary behaviors across different socioeconomic and cultural contexts.

The present systematic review also identified a variety of factors potentially influencing changes in adherence to a MD during the COVID-19 pandemic, pointing towards complex relationships and implications for public health. Seven studies showed that older age was associated with an increase in adherence, consistent with results from other studies (102). The three studies suggesting a decrease in adherence to a MD with older age all involved cohorts with a mean age above 65 years (65, 66, 70). This may imply an inverted U-shaped relationship, where adherence to a MD increases with age but declines after a certain age threshold, perhaps due to factors like limited access to appropriate dietary resources, physical restraints, and reduced independence to make one’s own choices (103, 104). Lower adherence to a MD has been associated with dementia, emphasizing the need for targeted nutritional support in older age groups (105).

This analysis additionally revealed that individuals with higher levels of education and secure socioeconomic and occupational status were more likely to adhere to a MD. These factors, combined with physical activity, lower body mass index, and residing in a family home point towards a profile of individuals who have more resources at their disposal and are able to maintain healthier lifestyles even during times of crises, which is in line with the findings from Alkerwi et al. (106). The decline in adherence to a MD during the pandemic, facilitated by factors such as reduced physical activity, increased rates of overweight/obesity, and heightened anxiety, has significant implications for future pandemics and global health crises. Irregular sleep, smoking, and the challenges of living in a city, being single, or caring for family members, indicate a far-reaching impact of the pandemic on public health. Public health policies addressing resource inequities, especially in underrepresented populations are urgently needed. To sustain public health during and in the aftermath of challenging times, it is crucial to provide comprehensive support that includes nutritional education, broad access to healthy foods to counteract food insecurity, the provision of mental health resources, and physical activity programs.

Several limitations should be considered when interpreting the findings of this systematic review. Questionnaire measures of adherence to a MD have the issue of their validity in common, as validation would require a direct observation of food intake to ensure their accuracy (107, 108). Furthermore, even though measurements of MD adherence may be good tools for assessing the desirable dietary behavior as part of the bigger picture (109), MD measures-especially short versions-may not fully capture nuanced changes in dietary patterns within populations. Furthermore, drawing conclusions for populations outside the Mediterranean region based solely on these data is challenging (107); therefore, further research is necessary to understand and validate the applicability of a MD in other populations as well. Additionally, only two studies included participants who identified their gender as different than men or women (57, 58), while they did not report the corresponding results. Consequently, sexual and gender minorities could not be considered adequately in this review. This oversight is significant, as research has shown that underrepresented groups were particularly impacted by the COVID-19 pandemic (110). Unique challenges and vulnerabilities, including greater psychological distress, economic instability, and limited access to healthcare and social support, necessitate tailored interventions. Future interventions should be specifically designed to support such high-risk groups, particularly during times of crisis.

Furthermore, the studies included in this synthesis are predominantly from the early phases of the pandemic (see Figure 2). There is a lack of data after 2021, which impedes drawing any conclusions about the long-term effects of the COVID-19 pandemic on adherence to a MD. Further investigations are necessary to address this gap. Finally, the quality of the included studies is another limitation; only five out of 29 studies met all quality criteria (4, 68, 80, 83, 84).

## 5 Conclusion

This systematic review aimed to examine gender differences in adherence to a MD during the COVID-19 pandemic and to identify factors influencing adherence. The findings revealed heterogeneous results: seven studies indicated higher adherence in men, 12 in women, and nine showed no significant gender differences. These inconsistencies may be attributed to differences in study sampling, the timing of data collection during the pandemic, and potentially further factors that require future investigation. Older age, higher education levels, higher socioeconomic status, and increased physical activity were associated with greater adherence to a MD. However, MD questionnaire measures may not fully capture nuanced changes in dietary patterns, while studies in populations outside the Mediterranean region are largely missing. In addition, the inclusion of participants who do not identify as a man or woman, or with sexual preferences other than heterosexual was effectively non-existent, thus preventing an adequate consideration of SGM individuals. In conclusion, while this systematic review provides insights into gender differences in MD adherence during the COVID-19 pandemic, it underscores the need for more representative and longitudinal research. Future studies should consider diverse populations, including SGM, and examine long-term dietary patterns to develop targeted nutritional interventions during crises.

## Data Availability

The original contributions presented in the study are included in the article/Supplementary material, further inquiries can be directed to the corresponding author.

## References

[1] World Health Organization. (2024). WHO COVID-19 dashboard. Available at: https://data.who.int/dashboards/covid19/cases?n=c

[2] Hu B Guo H Zhou P Shi Z-L. Characteristics of SARS-CoV-2 and COVID-19. Nat Rev Microbiol. (2021) 19:141–54. doi: 10.1038/s41579-020-00459-7 33024307 PMC7537588

[3] Talic S Shah S Wild H Gasevic D Maharaj A Ademi Z . Effectiveness of public health measures in reducing the incidence of COVID-19, SARS-CoV-2 transmission, and COVID-19 mortality: systematic review and meta-analysis. BMJ. (2021):e068302. doi: 10.1136/bmj-2021-068302 34789505 PMC9423125

[4] Sánchez-Sánchez E Ramírez-Vargas G Avellaneda-López Y Orellana-Pecino JI García-Marín E Díaz-Jimenez J. Eating habits and physical activity of the Spanish population during the COVID-19 pandemic period. Nutrients. (2020) 12:2826. doi: 10.3390/nu12092826 32942695 PMC7551353

[5] Di Renzo L Gualtieri P Pivari F Soldati L Attinà A Cinelli G . Eating habits and lifestyle changes during COVID-19 lockdown: an Italian survey. J Transl Med. (2020) 18:229. doi: 10.1186/s12967-020-02399-5 32513197 PMC7278251

[6] Yannakoulia M Panagiotakos DB Pitsavos C Tsetsekou E Fappa E Papageorgiou C . Eating habits in relations to anxiety symptoms among apparently healthy adults. A pattern analysis from the ATTICA study. Appetite. (2008) 51:519–25. doi: 10.1016/j.appet.2008.04.002 18495296

[7] Mattioli AV Ballerini Puviani M Nasi M Farinetti A. COVID-19 pandemic: the effects of quarantine on cardiovascular risk. Eur J Clin Nutr. (2020) 74:852–5. doi: 10.1038/s41430-020-0646-z 32371988 PMC7199203

[8] Butler MJ Barrientos RM. The impact of nutrition on COVID-19 susceptibility and long-term consequences. Brain Behav Immun. (2020) 87:53–4. doi: 10.1016/j.bbi.2020.04.040 32311498 PMC7165103

[9] Hendren NS de Lemos JA Ayers C Das SR Rao A Carter S . Association of body mass index and age with morbidity and mortality in patients hospitalized with COVID-19. Circulation. (2021) 143:135–44. doi: 10.1161/CIRCULATIONAHA.120.051936 33200947

[10] Milton-Laskibar I Trepiana J Macarulla MT Gómez-Zorita S Arellano-García L Fernández-Quintela A . Potential usefulness of Mediterranean diet polyphenols against COVID-19-induced inflammation: a review of the current knowledge. J Physiol Biochem. (2023) 79:371–82. doi: 10.1007/s13105-022-00926-0 36346507 PMC9641689

[11] Angelidi AM Kokkinos A Katechaki E Ros E Mantzoros CS. Mediterranean diet as a nutritional approach for COVID-19. Metabolism. (2021) 114:154407. doi: 10.1016/j.metabol.2020.154407 33080270 PMC7833284

[12] Popkin BM Du S Green WD Beck MA Algaith T Herbst CH . Individuals with obesity and COVID-19: a global perspective on the epidemiology and biological relationships. Obes Rev. (2020) 21:e13128. doi: 10.1111/obr.13128 32845580 PMC7461480

[13] Vitale E Magrone M Galatola V Magrone T. The role of nutrition during the COVID-19 pandemic: what we know. Endocr Metab Immune Disord Drug Targets. (2021) 21:1982–92. doi: 10.2174/1871530321666210114154401 33459251

[14] Wu D Lewis ED Pae M Meydani SN. Nutritional modulation of immune function: analysis of evidence, mechanisms, and clinical relevance. Front Immunol. (2019) 9:3160. doi: 10.3389/fimmu.2018.03160 30697214 PMC6340979

[15] Willett W Sacks F Trichopoulou A Drescher G Ferro-Luzzi A Helsing E . Mediterranean diet pyramid: a cultural model for healthy eating. Am J Clin Nutr. (1995) 61:1402S–6S. doi: 10.1093/ajcn/61.6.1402S 7754995

[16] Dinu M Pagliai G Casini A Sofi F. Mediterranean diet and multiple health outcomes: an umbrella review of meta-analyses of observational studies and randomised trials. Eur J Clin Nutr. (2018) 72:30–43. doi: 10.1038/ejcn.2017.58 28488692

[17] Rosato V Temple NJ La Vecchia C Castellan G Tavani A Guercio V. Mediterranean diet and cardiovascular disease: a systematic review and meta-analysis of observational studies. Eur J Nutr. (2019) 58:173–91. doi: 10.1007/s00394-017-1582-0 29177567

[18] Galbete C Schwingshackl L Schwedhelm C Boeing H Schulze MB. Evaluating Mediterranean diet and risk of chronic disease in cohort studies: an umbrella review of meta-analyses. Eur J Epidemiol. (2018) 33:909–31. doi: 10.1007/s10654-018-0427-3 30030684 PMC6153506

[19] Barrea L Muscogiuri G Frias-Toral E Laudisio D Pugliese G Castellucci B . Nutrition and immune system: from the Mediterranean diet to dietary supplementary through the microbiota. Crit Rev Food Sci Nutr. (2021) 61:3066–90. doi: 10.1080/10408398.2020.1792826 32691606

[20] Scoditti E Capurso C Capurso A Massaro M. Vascular effects of the Mediterranean diet—part II: role of omega-3 fatty acids and olive oil polyphenols. Vasc Pharmacol. (2014) 63:127–34. doi: 10.1016/j.vph.2014.07.001 25446163

[21] Ventriglio A Sancassiani F Contu MP Latorre M Di Slavatore M Fornaro M . Mediterranean diet and its benefits on health and mental health: a literature review. Clin Pract Epidemiol Ment Health. (2020) 16:156–64. doi: 10.2174/1745017902016010156 33029192 PMC7536728

[22] Estruch R Ros E Salas-Salvadó J Covas M-I Corella D Arós F . Primary prevention of cardiovascular disease with a Mediterranean diet supplemented with extra-virgin olive oil or nuts. N Engl J Med. (2018) 378:e34. doi: 10.1056/NEJMoa1800389 29897866

[23] Bucciantini M Leri M Nardiello P Casamenti F Stefani M. Olive polyphenols: antioxidant and anti-inflammatory properties. Antioxidants. (2021) 10:1044. doi: 10.3390/antiox10071044 34209636 PMC8300823

[24] Hidalgo-Liberona N Meroño T Zamora-Ros R Rabassa M Semba R Tanaka T . Adherence to the Mediterranean diet assessed by a novel dietary biomarker score and mortality in older adults: the InCHIANTI cohort study. BMC Med. (2021) 19:280. doi: 10.1186/s12916-021-02154-7 34814922 PMC8611910

[25] Di Nucci A Silano M Cardamone E. Adherence to Mediterranean diet and health outcomes in adolescents: an umbrella review. Nutr Rev. (2024). doi: 10.1093/nutrit/nuae085 PMC11819493 38954538

[26] García-Montero C Fraile-Martínez O Gómez-Lahoz AM Pekarek L Castellanos AJ Noguerales-Fraguas F . Nutritional components in western diet versus Mediterranean diet at the gut microbiota-immune system interplay. Implications for health and disease. Nutrients. (2021) 13:699. doi: 10.3390/nu13020699 33671569 PMC7927055

[27] Bonaccio M Iacoviello L de Gaetano G Moli-Sani Investigators. The Mediterranean diet: the reasons for a success. Thromb Res. (2012) 129:401–4. doi: 10.1016/j.thromres.2011.10.018 22100317

[28] Kyriacou A Evans JMM Economides N Kyriacou A. Adherence to the Mediterranean diet by the Greek and Cypriot population: a systematic review. Eur J Pub Health. (2015) 25:1012–8. doi: 10.1093/eurpub/ckv124 26130797

[29] Halton TL Willett WC Liu S Manson JE Stampfer MJ Hu FB. Potato and french fry consumption and risk of type 2 diabetes in women. Am J Clin Nutr. (2006) 83:284–90. doi: 10.1093/ajcn/83.2.284 16469985

[30] da Costa GG da Conceição Nepomuceno G da Silva Pereira A Simões BFT. Worldwide dietary patterns and their association with socioeconomic data: an ecological exploratory study. Glob Health. (2022) 18:31. doi: 10.1186/s12992-022-00820-w PMC8917745 35279165

[31] Garcia M Mulvagh SL Bairey Merz CN Buring JE Manson JE. Cardiovascular disease in women. Circ Res. (2016) 118:1273–93. doi: 10.1161/CIRCRESAHA.116.307547 27081110 PMC4834856

[32] Bates N Chin M Becker T eds. Measuring sex, gender identity, and sexual orientation. Washington, DC: National Academies Press (2022). 35286054

[33] Schorr M Dichtel LE Gerweck AV Valera RD Torriani M Miller KK . Sex differences in body composition and association with cardiometabolic risk. Biol Sex Differ. (2018) 9:28. doi: 10.1186/s13293-018-0189-3 29950175 PMC6022328

[34] Eaton SA Sethi JK. Immunometabolic links between estrogen, adipose tissue and female reproductive metabolism. Biology. (2019) 8:8. doi: 10.3390/biology8010008 30736459 PMC6466614

[35] Tramunt B Smati S Grandgeorge N Lenfant F Arnal J-F Montagner A . Sex differences in metabolic regulation and diabetes susceptibility. Diabetologia. (2020) 63:453–61. doi: 10.1007/s00125-019-05040-3 31754750 PMC6997275

[36] Kau AL Ahern PP Griffin NW Goodman AL Gordon JI. Human nutrition, the gut microbiome and the immune system. Nature. (2011) 474:327–36. doi: 10.1038/nature10213 21677749 PMC3298082

[37] Dominianni C Sinha R Goedert JJ Pei Z Yang L Hayes RB . Sex, body mass index, and dietary fiber intake influence the human gut microbiome. PLoS One. (2015) 10:e0124599. doi: 10.1371/journal.pone.0124599 25874569 PMC4398427

[38] Pant A Chew D Mamas M Zaman S. Cardiovascular disease and the Mediterranean diet: insights into sex-specific responses. Nutrients. (2024) 16:570. doi: 10.3390/nu16040570 38398894 PMC10893368

[39] Barrea L Verde L Suárez R Frias-Toral E Vásquez CA Colao A . Sex-differences in Mediterranean diet: a key piece to explain sex-related cardiovascular risk in obesity? A cross-sectional study. J Transl Med. (2024) 22:44. doi: 10.1186/s12967-023-04814-z 38200498 PMC10782790

[40] Delgado-Lista J Alcala-Diaz JF Torres-Peña JD Quintana-Navarro GM Fuentes F Garcia-Rios A . Long-term secondary prevention of cardiovascular disease with a Mediterranean diet and a low-fat diet (CORDIOPREV): a randomised controlled trial. Lancet. (2022) 399:1876–85. doi: 10.1016/S0140-6736(22)00122-2 35525255

[41] Grzymisławska M Puch E Zawada A Grzymisławski M. Do nutritional behaviors depend on biological sex and cultural gender? Adv Clin Exp Med. (2020) 29:165–72. doi: 10.17219/acem/111817 32017478

[42] Lombardo M Aulisa G Padua E Annino G Iellamo F Pratesi A . Gender differences in taste and foods habits. Nutr Food Sci. (2019) 50:229–39. doi: 10.1108/NFS-04-2019-0132

[43] Feraco A Armani A Amoah I Guseva E Camajani E Gorini S . Assessing gender differences in food preferences and physical activity: a population-based survey. Front Nutr. (2024) 11:1348456. doi: 10.3389/fnut.2024.1348456 38445208 PMC10912473

[44] Wardle J Haase AM Steptoe A Nillapun M Jonwutiwes K Bellisie F. Gender differences in food choice: the contribution of health beliefs and dieting. Ann Behav Med. (2004) 27:107–16. doi: 10.1207/s15324796abm2702_5 15053018

[45] Arvaniti F Panagiotakos DB Pitsavos C Zampelas A Stefanadis C. Dietary habits in a Greek sample of men and women: the ATTICA study. Cent Eur J Public Health. (2006) 14:74–7. doi: 10.21101/cejph.a3374 16830608

[46] Obeid CA Gubbels JS Jaalouk D Kremers SPJ Oenema A. Adherence to the Mediterranean diet among adults in Mediterranean countries: a systematic literature review. Eur J Nutr. (2022) 61:3327–44. doi: 10.1007/s00394-022-02885-0 35451614 PMC9026058

[47] Moore E Fadel A Lane KE. The effects of consuming a Mediterranean style diet on associated COVID-19 severity biomarkers in obese/overweight adults: a systematic review. Nutr Health. (2022) 28:647–67. doi: 10.1177/02601060221127853 36131504 PMC9494166

[48] Della Valle PG Mosconi G Nucci D Pietro VG Gentile L Gianfredi V . Adherence to the Mediterranean diet during the COVID-19 national lockdowns: a systematic review of observational studies. Acta Biomed. (2021) 92:e2021440. doi: 10.23750/abm.v92iS6.12233 34739464 PMC8851000

[49] González-Monroy C Gómez-Gómez I Olarte-Sánchez CM Motrico E. Eating behaviour changes during the COVID-19 pandemic: a systematic review of longitudinal studies. Int J Environ Res Public Health. (2021) 18:11130. doi: 10.3390/ijerph182111130 34769648 PMC8582896

[50] Schiavo JH. PROSPERO: an international register of systematic review protocols. Med Ref Serv Q. (2019) 38:171–80. doi: 10.1080/02763869.2019.1588072 31173570

[51] Moher D. Preferred reporting items for systematic reviews and Meta-analyses: the PRISMA statement. Ann Intern Med. (2009) 151:264. doi: 10.7326/0003-4819-151-4-200908180-00135 19622511

[52] Veritas Health Innovation. Covidence. Melbourne, VIC: Veritas Health Innovation (2024).

[53] Ma L-L Wang Y-Y Yang Z-H Huang D Weng H Zeng X-T. Methodological quality (risk of bias) assessment tools for primary and secondary medical studies: what are they and which is better? Mil Med Res. (2020) 7:7. doi: 10.1186/s40779-020-00238-8 32111253 PMC7049186

[54] The Joanna Briggs Institute. Critical appraisal checklist for analytical cross sectional studies In: Checklist for analytical cross sectional studies. Adelaide, SA: The Joanna Briggs Institute (2017)

[55] Costarelli V Michou M. Predictors of COVID-19 vaccine hesitancy and prevention practice in Greece. Int J Health Promot Educ. (2022) 62:98–113. doi: 10.1080/14635240.2022.2073554

[56] Costarelli V Michou M. Perceived stress negatively affects diet quality and life satisfaction during the COVID-19 lockdown period, in Greece. Nutr Food Sci. (2023) 53:769–80. doi: 10.1108/NFS-12-2022-0403

[57] Sulejmani E Hyseni A Xhabiri G Rodríguez-Pérez C. Relationship in dietary habits variations during COVID-19 lockdown in Kosovo: the COVIDiet study. Appetite. (2021) 164:105244. doi: 10.1016/j.appet.2021.105244 33848591 PMC8035802

[58] Giacalone D Frøst MB Rodríguez-Pérez C. Reported changes in dietary habits during the COVID-19 lockdown in the Danish population: the Danish COVIDiet study. Front Nutr. (2020) 7:592112. doi: 10.3389/fnut.2020.592112 33364250 PMC7752855

[59] García-Campanario I Viñolo Gil MJ Vanlinthout LE Pérez Pérez C O’Ferrall GC. Gender differences regarding self-perceived physical and mental health in Spanish university sports and physical therapy students after termination of the COVID-19 lockdown period. Healthcare. (2024) 12:191. doi: 10.3390/healthcare12020191 38255079 PMC10815373

[60] Schröder H Fitó M Estruch R Martínez-González MA Corella D Salas-Salvadó J . A short screener is valid for assessing Mediterranean diet adherence among older Spanish men and women. J Nutr. (2011) 141:1140–5. doi: 10.3945/jn.110.135566 21508208

[61] Martínez-González MA García-Arellano A Toledo E Salas-Salvadó J Buil-Cosiales P Corella D . A 14-item Mediterranean diet assessment tool and obesity indexes among high-risk subjects: the PREDIMED trial. PLoS One. (2012) 7:e43134. doi: 10.1371/journal.pone.0043134 22905215 PMC3419206

[62] Panagiotakos DB Pitsavos C Stefanadis C. Dietary patterns: a Mediterranean diet score and its relation to clinical and biological markers of cardiovascular disease risk. Nutr Metab Cardiovasc Dis. (2006) 16:559–68. doi: 10.1016/j.numecd.2005.08.006 17126772

[63] Trichopoulou A Costacou T Bamia C Trichopoulos D. Adherence to a Mediterranean diet and survival in a Greek population. N Engl J Med. (2003) 348:2599–608. doi: 10.1056/NEJMoa025039 12826634

[64] Serra-Majem L Ribas L Ngo J Ortega RM García A Pérez-Rodrigo C . Food, youth and the Mediterranean diet in Spain. Development of KIDMED, Mediterranean Diet Quality Index in children and adolescents. Public Health Nutr. (2004) 7:931–5. doi: 10.1079/PHN2004556 15482620

[65] Bonaccio M Gianfagna F Stival C Amerio A Bosetti C D’oro LC . Changes in a Mediterranean lifestyle during the COVID-19 pandemic among elderly Italians: an analysis of gender and socioeconomic inequalities in the “LOST in Lombardia” study. Int J Food Sci Nutr. (2022) 73:683–92. doi: 10.1080/09637486.2022.2040009 35285380

[66] Carbayo Herencia JA Rosich N Panisello Royo JM Carro A Allins Presas J Panisello M . Influence of the confinement that occurred in Spain due to the SARS-CoV-2 virus outbreak on adherence to the Mediterranean diet. Clin Investig Arterioscler. (2021) 33:235–46. doi: 10.1016/j.arteri.2021.01.005 PMC7883718 34092432

[67] Karam J Ghach W Bouteen C Makary MJ Riman M Serhan M. Adherence to Mediterranean diet among adults during the COVID-19 outbreak and the economic crisis in Lebanon. Nutr Food Sci. (2022) 52:1018–28. doi: 10.1108/NFS-10-2021-0325

[68] Kyprianidou M Christophi CA Giannakou K. Quarantine during COVID-19 outbreak: adherence to the Mediterranean diet among the Cypriot population. Nutrition. (2021) 90:111313. doi: 10.1016/j.nut.2021.111313 34119718 PMC9759705

[69] Mascherini G Catelan D Pellegrini-Giampietro DE Petri C Scaletti C Gulisano M. Changes in physical activity levels, eating habits and psychological well-being during the Italian COVID-19 pandemic lockdown: impact of socio-demographic factors on the Florentine academic population. PLoS One. (2021) 16:e0252395. doi: 10.1371/journal.pone.0252395 34043739 PMC8159001

[70] Mattavelli E Olmastroni E Casula M Grigore L Pellegatta F Baragetti A . Adherence to Mediterranean diet: a population-based longitudinal cohort study. Nutrients. (2023) 15:1844. doi: 10.3390/nu15081844 37111063 PMC10145158

[71] Sumalla-Cano S Forbes-Hernández T Aparicio-Obregón S Crespo J Eléxpuru-Zabaleta M Gracia-Villar M . Changes in the lifestyle of the Spanish university population during confinement for COVID-19. Int J Environ Res Public Health. (2022) 19:2210. doi: 10.3390/ijerph19042210 35206397 PMC8872173

[72] Scoditti E Bodini A Sabina S Leo CG Mincarone P Rissotto A . Effects of working from home on lifestyle behaviors and mental health during the COVID-19 pandemic: a survey study. PLoS One. (2024) 19:e0300812. doi: 10.1371/journal.pone.0300812 38558099 PMC10984516

[73] Gualtieri P Frank G Cianci R Smeriglio A Alibrandi A Di Renzo L . Mediterranean diet influence on SARS-CoV-2 vaccine adverse reaction: friend or foe? Nutrients. (2024) 16:1846. doi: 10.3390/nu16121846 38931201 PMC11206327

[74] Doğan G Özyildirim C Yabanci AN. Supplementation use and diet changes during COVID-19 pandemic according to anxiety level and Mediterranean diet adherence. Clin Nutr ESPEN. (2023) 54:122–9. doi: 10.1016/j.clnesp.2023.01.022 36963853 PMC9873361

[75] Gumus D Topal GG Sevim S Kizil M. Adherence to Mediterranean diet and dietary changes according to the fear of COVID-19 during the pandemic: a cross-sectional study. J Nutr Sci. (2023) 12:e56. doi: 10.1017/jns.2023.40 37180483 PMC10173089

[76] Kocaay F Ayyildiz P Şanlier N. Enquiring into experiences of fear, posttraumatic stress and nutritional habits of medical students during the COVID-19 pandemic. Duzce Med J. (2022) 24:307–14. doi: 10.18678/dtfd.1192388

[77] Rodríguez-Pérez C Molina-Montes E Verardo V Artacho R García-Villanova B Guerra-Hernández EJ . Changes in dietary behaviours during the COVID-19 outbreak confinement in the Spanish COVIDiet study. Nutrients. (2020) 12:1730. doi: 10.3390/nu12061730 32531892 PMC7353108

[78] Pavlidou E Papadopoulou SK Mentzelou M Dakanalis A Vorvolakos T Antasouras G . Association of Mediterranean diet adherence with sociodemographic, anthropometric, and lifestyle factors during the COVID-19 pandemic: a cross-sectional study in Greece. Nutrients. (2023) 15:4123. doi: 10.3390/nu15194123 37836406 PMC10574046

[79] Atabilen B Akbulut G Koçak T Tek N. Evaluation of emotional state and Mediterranean diet adherence during the COVID-19 pandemic: butterfly effect. Clin Exp Health Sci. (2023) 13:323–9. doi: 10.33808/clinexphealthsci.1105236

[80] Ejder ZB Sanlier N. Perceived impact of quarantine period on food craving, power of food, and the Mediterranean diet: the dark side of pandemic fear. Int J Gastron Food Sci. (2023) 32:100689. doi: 10.1016/j.ijgfs.2023.100689

[81] García-Esquinas E Ortolá R Gine-Vázquez I Carnicero JA Mañas A Lara E . Changes in health behaviors, mental and physical health among older adults under severe lockdown restrictions during the COVID-19 pandemic in Spain. Int J Environ Res Public Health. (2021) 18:7067. doi: 10.3390/ijerph18137067 34281004 PMC8297096

[82] Mulè A Galasso L Castelli L Ciorciari A Michielon G Esposito F . Lifestyle of Italian university students attending different degree courses: a survey on physical activity, sleep and eating behaviors during the COVID-19 pandemic. Sustainability. (2022) 14:15340. doi: 10.3390/su142215340

[83] Perez-Araluce R Martinez-Gonzalez MA Fernández-Lázaro CI Bes-Rastrollo M Gea A Carlos S. Mediterranean diet and the risk of COVID-19 in the ‘Seguimiento Universidad de Navarra’ cohort. Clin Nutr. (2022) 41:3061–8. doi: 10.1016/j.clnu.2021.04.001 33934925 PMC8047333

[84] Turki S Bouzekri K Trabelsi T El Ati J. Assessment of Mediterranean diet adherence and lifestyle change during COVID-19 national lockdown in Tunisian adult population. Nutrients. (2022) 14:4151. doi: 10.3390/nu14194151 36235802 PMC9572866

[85] Saleh MR Abdelgaied MY Galal N Tarek M Fouda A Abdelkawy K. Unveiling the lockdown effects: exploring behavior, dietary habits and weight changes in rural Egypt during COVID-19 lockdown: a cross-sectional retrospective study. J Health Popul Nutr. (2024) 43:85. doi: 10.1186/s41043-024-00558-8 38879511 PMC11179345

[86] Abreu F Hernando A Goulão LF Pinto AM Branco A Cerqueira A . Mediterranean diet adherence and nutritional literacy: an observational cross-sectional study of the reality of university students in a COVID-19 pandemic context. BMJ Nutr Prev Health. (2023) 6:221–30. doi: 10.1136/bmjnph-2023-000659 PMC10862292 38357557

[87] Abdelkawy K Elbarbry F El-masry SM Zakaria AY Rodríguez-Pérez C El-khodary NM. Changes in dietary habits during COVID-19 lockdown in Egypt: the Egyptian COVIDiet study. BMC Public Health. (2023) 23:956. doi: 10.1186/s12889-023-15777-7 37231373 PMC10209922

[88] Wild H Kyröläinen A-J Kuperman V. How representative are student convenience samples? A study of literacy and numeracy skills in 32 countries. PLoS One. (2022) 17:e0271191. doi: 10.1371/journal.pone.0271191 35802736 PMC9269910

[89] Carli LL. Women, gender equality and COVID-19. Gend Manag Int J. (2020) 35:647–55. doi: 10.1108/GM-07-2020-0236

[90] Matud MP. Gender differences in stress and coping styles. Pers Individ Differ. (2004) 37:1401–15. doi: 10.1016/j.paid.2004.01.010

[91] Prowse R Sherratt F Abizaid A Gabrys RL Hellemans KGC Patterson ZR . Coping with the COVID-19 pandemic: examining gender differences in stress and mental health among university students. Front Psychiatry. (2021) 12:650759. doi: 10.3389/fpsyt.2021.650759 33897499 PMC8058407

[92] Barcın-Güzeldere HK Devrim-Lanpir A. The association between body mass index, emotional eating and perceived stress during COVID-19 partial quarantine in healthy adults. Public Health Nutr. (2022) 25:43–50. doi: 10.1017/S1368980021002974 34261563 PMC8365042

[93] Kaner G Yurtdaş-Depboylu G Çalık G Yalçın T Nalçakan T. Evaluation of perceived depression, anxiety, stress levels and emotional eating behaviours and their predictors among adults during the COVID-19 pandemic. Public Health Nutr. (2023) 26:674–83. doi: 10.1017/S1368980022002579 36453207 PMC9767902

[94] Bhattacharjee A Ghosh T. COVID-19 pandemic and stress: coping with the new Normal. J Prev Health Promot. (2022) 3:30–52. doi: 10.1177/26320770211050058 35194577 PMC8855221

[95] Altman DG Bland JM. Statistics notes: absence of evidence is not evidence of absence. BMJ. (1995) 311:485–5. doi: 10.1136/bmj.311.7003.485 7647644 PMC2550545

[96] Mendonça N Gregório MJ Salvador C Henriques AR Canhão H Rodrigues AM. Low adherence to the Mediterranean diet is associated with poor socioeconomic status and younger age: a cross-sectional analysis of the EpiDoC cohort. Nutrients. (2022) 14:1239. doi: 10.3390/nu14061239 35334895 PMC8954252

[97] Velhinho AR Perelman J. Socioeconomic inequalities in food consumption: a cross-sectional study in Portuguese adults. Port J Public Health. (2021) 39:11–20. doi: 10.1159/000515937 39469036 PMC11320100

[98] Metin ZE Çelik ÖM Koç N. Relationship between adherence to the Mediterranean diet, sustainable and healthy eating behaviors, and climate change awareness: a cross-sectional study from Turkey. Nutrition. (2024) 118:112266. doi: 10.1016/j.nut.2023.112266 37988926

[99] Aytekin Sahin G Mengi CO. Evaluation of food insecurity and associated factors in women of childbearing age: a community-based study from Turkey. Food Sci Nutr. (2024) 12:154–61. doi: 10.1002/fsn3.3743 38323300 PMC10846561

[100] Das S Mishra AJ. Dietary practices and gender dynamics: understanding the role of women. J Ethn Foods. (2021) 8:4. doi: 10.1186/s42779-021-00081-9

[101] IMF. (2024). Internation monetary fund: Datamapper 2024. Available at: https://www.imf.org/external/datamapper/NGDPDPC@WEO/ESP/ITA/TUR/EGY/GRC/CYP/DNK/UVK/LBN/PRT/TUN

[102] Patino-Alonso MC Recio-Rodríguez JI Belio JFM Colominas-Garrido R Lema-Bartolomé J Arranz AG . Factors associated with adherence to the Mediterranean diet in the adult population. J Acad Nutr Diet. (2014) 114:583–9. doi: 10.1016/j.jand.2013.07.038 24209889

[103] Mavegam Tango Assoumou BO Coughenour C Godbole A McDonough I. Senior food insecurity in the USA: a systematic literature review. Public Health Nutr. (2023) 26:229–45. doi: 10.1017/S1368980022002415 36329645 PMC11077460

[104] Pereira MHQ Pereira MLAS Campos GC Molina MCB. Food insecurity and nutritional status among older adults: a systematic review. Nutr Rev. (2022) 80:631–44. doi: 10.1093/nutrit/nuab044 34338784

[105] Nicoli C Galbussera AA Bosetti C Franchi C Gallus S Mandelli S . The role of diet on the risk of dementia in the oldest old: the Monzino 80-plus population-based study. Clin Nutr. (2021) 40:4783–91. doi: 10.1016/j.clnu.2021.06.016 34242918

[106] Alkerwi A Vernier C Sauvageot N Crichton GE Elias MF. Demographic and socioeconomic disparity in nutrition: application of a novel correlated component regression approach. BMJ Open. (2015) 5:e006814. doi: 10.1136/bmjopen-2014-006814 PMC4431064 25967988

[107] Hutchins-Wiese HL Bales CW Porter Starr KN. Mediterranean diet scoring systems: understanding the evolution and applications for Mediterranean and non-Mediterranean countries. Br J Nutr. (2022) 128:1371–92. doi: 10.1017/S0007114521002476 34289917

[108] Zaragoza-Martí A Cabañero-Martínez M Hurtado-Sánchez J Laguna-Pérez A Ferrer-Cascales R. Evaluation of Mediterranean diet adherence scores: a systematic review. BMJ Open. (2018) 8:e019033. doi: 10.1136/bmjopen-2017-019033 PMC5855302 29478018

[109] Miller V Webb P Micha R Mozaffarian D. Defining diet quality: a synthesis of dietary quality metrics and their validity for the double burden of malnutrition. Lancet Planet Health. (2020) 4:e352–70. doi: 10.1016/S2542-5196(20)30162-5 32800153 PMC7435701

[110] Laskowski NM Brandt G Paslakis G. Geschlechtsspezifische Unterschiede und Ungleichheiten der COVID-19 Pandemie: Eine Synthese systematischer Reviews unter Einbeziehung sexueller und geschlechtlicher Minderheiten. Psychother Psychosom Med Psychol. (2024) 74:57–69. doi: 10.1055/a-2228-6244 38316434

