# Supplementary material for: Gender and sex differences in adherence to a Mediterranean diet and associated factors during the COVID-19 pandemic: a systematic review
Source: Front Nutr. 2025 Jan 7;11:1501646. doi: 10.3389/fnut.2024.1501646 (PMC11784154; doi:10.3389/fnut.2024.1501646)
Supplement: Supplementary file 1 [file Table_1.DOCX]

Supplementary Material

# Supplementary Table

**Search Strategy of All Databases**

| **Database** | **Search Query** |
| --- | --- |
| **PubMed** | ((diet[Title/Abstract]) OR (nutrition[Title/Abstract]) OR (binge*[Title/Abstract]) OR (eating behavio*[Title/Abstract]) OR (eating habit*[Title/Abstract]) OR (eating disorder*[Title/Abstract]) OR (disordered eating[Title/Abstract]) OR (body *satisfaction[Title/Abstract]) OR (body image[Title/Abstract])) AND ((gender*[Title/Abstract]) OR (sex[Title/Abstract]) OR (men[Title/Abstract]) OR (women[Title/Abstract]) OR (trans*[Title/Abstract]) OR (lgb[Title/Abstract]) OR (lgbt*[Title/Abstract]) OR (intersex*[Title/Abstract]) OR (*binary[Title/Abstract]) OR (queer[Title/Abstract]) OR (male[Title/Abstract]) OR (female[Title/Abstract])) AND ((sarscov[Title/Abstract]) OR (pandem*[Title/Abstract]) OR (COVID-19[Title/Abstract]) OR (corona*[Title/Abstract]) OR (lockdown[Title/Abstract]) AND ((English[Language]) OR (German[Language])) AND (2019:2023[pdat])) |
| **Web Of Science** | ((TS=(“diet” OR “nutrition” OR “binge*” OR “eating behavio*” OR “eating habit*” OR “eating disorder*” OR “disordered eating” OR “body dissatisfaction” OR “body satisfaction” OR “body image”)) AND (TS=(“gender*” OR “sex” OR “men” OR “women” OR “trans*” OR “LGB” OR “LGBT*” OR “intersex*” OR “nonbinary” OR “non-binary” OR “queer” OR “male” OR “female”)) AND (TS=(“sars-cov” OR “pandem*” OR “COVID-19” OR “corona*” OR “lockdown”)) AND (LA=(English OR German)) AND (PY=(2019-2023))) |
| **PsycInfo** | ((“diet” OR “nutrition” OR “binge*” OR “eating behavio*” OR “eating habit*” OR “eating disorder*” OR “disordered eating” OR “body image” OR “body dissatisfaction” OR “body satisfaction”) AND (“gender*” OR “sex” OR “men” OR “women” OR “trans*” OR “LGB” OR “LGBT*” OR “intersex*” OR “nonbinary” OR “non-binary” OR “queer” OR “male” OR “female”) AND (“sars-cov” OR “pandem*” OR “COVID-19” OR “corona*” OR lockdown”) AND (DT 2019-2023) AND (LA (english or german))) |
| **Scopus** | (TITLE-ABS-KEY({diet}) OR TITLE-ABS-KEY({nutrition}) OR TITLE-ABSKEY({binge*}) OR TITLE-ABS-KEY({eating behavio*}) OR TITLE-ABS-KEY({eating habit*}) OR TITLE-ABS-KEY({eating disorder*}) OR TITLE-ABS-KEY({disordered eating}) OR TITLE-ABS-KEY({body image}) OR TITLE-ABS-KEY({body dissatisfaction}) OR TITLE-ABS-KEY({body satisfaction})) AND (TITLE-ABSKEY({gender*}) OR TITLE-ABS-KEY({sex}) OR TITLE-ABS-KEY({men}) OR TITLEABS-KEY({women}) OR TITLE-ABS-KEY({trans*}) OR TITLE-ABS-KEY({LGB}) OR TITLE-ABS-KEY({LGBT*}) OR TITLE-ABS-KEY({intersex*}) OR TITLE-ABSKEY({nonbinary}) OR TITLE-ABS-KEY({non-binary}) OR TITLE-ABS-KEY({queer}) OR TITLE-ABS-KEY({male}) OR TITLE-ABS-KEY({female})) AND (TITLE-ABSKEY({sars-cov}) OR TITLE-ABS-KEY({pandem*}) OR TITLE-ABS-KEY({COVID-19}) OR TITLE-ABS-KEY({corona*}) OR TITLE-ABS-KEY({lockdown})) AND (PUBYEAR > 2019) |
